# Mongolian pine forest decline by the combinatory effect of European woodwasp and plant pathogenic fungi

**DOI:** 10.1038/s41598-021-98795-y

**Published:** 2021-10-04

**Authors:** Lixiang Wang, Chunchun Li, Youqing Luo, Lili Ren, Ning Lv, Jing-Jiang Zhou, Senshan Wang

**Affiliations:** 1 Biocontrol Engineering Laboratory of Crop Diseases and Pests of Gansu Province, College of Plant Protection, Gansu Agricultural University, Lanzhou, 730070 China; 2 Beijing Key Laboratory for Forest Pest Control, Beijing Forestry University, Beijing, 100083 China; 3 Sino-France Joint Laboratory for Invasive Forest Pests in Eurasia, Beijing, 100083 China; 4 State Key Laboratory of Green Pesticide and Agricultural Bioengineering, Ministry of Education, Guizhou University, Huaxi District, Guiyang, 550025 China

**Keywords:** Forest ecology, Forestry, Microbial ecology

## Abstract

Interactions between the decline of Mongolian pine woodlands and fungal communities and invasive pests in northeastern China are poorly understood. In this study, we investigated the fungal communities occurring in three tree samples: the woodwasp *Sirex noctilio* infested, healthy uninfested and unhealthy uninfested Mongolian pine trees. We analyzed the relationships of the Mongolian pine decline with fungal infection and woodwasp infestation. Twenty-six fungal species were identified from the sampled trees. Each tree sample harbored a fungal endophyte community with a unique structure. Pathogenic fungi richness was four times higher in infested and unhealthy un-infested trees compared to that in healthy uninfested trees. *Sphaeropsis sapinea* was the most dominant pathogenic fungus in the sampled Mongolian pine trees. The number of *S. noctilio* was higher than native bark beetles in the declining Mongolian pine trees. The invasion of the woodwasp appeared to be promoted by the fungal infection in the Mongolian pine trees. The incidence of *S. noctilio* infestation was higher in the fungi infected trees (83.22%) than those without infection (38.72%). *S. sapinea* population exhibited positive associations with within-tree colonization of *S. noctilio* and bark beetle. Collectively, these data indicate that the fungal disease may have caused as the initial reason the decline of the Mongolian pine trees, and also provided convenient conditions for the successful colonization of the woodwasp. The woodwasps attack the Mongolian pine trees infected by fungi and accelerated its decline.

## Introduction

Mongolian pine (*Pinus sylvestris* var. *mongolica*), a geographical variety of Scots pine (*P. sylvestris*), is naturally distributed in the Daxinganling mountains of China, in Honghuaerji of the Hulunbeier sandy plains of China, and in parts of Russia and Mongolia. It is often planted as an ornamental tree because of its height and greening characteristics. Also, this tree is characterized by cold hardiness, drought tolerance, strong adaptability and rapid growth^1,2^. It is currently the main coniferous tree species utilized in the “3-North Shelter Forest Program” and the “Sand-Control Project” in China and plays an important role in ecological construction and environmental restoration^3^.

Over the last decades, as the area of Mongolian pine plantations grows year by year, a widespread decline phenomena and extensive mortality events of the Mongolian pine forest have been observed in several parts of China, revealing the high vulnerability of these forests to fungi infection and pest infestation^4^. Severe decline and mortality events have the potential to drastically alter Mongolian pine ecosystems, with important implications for the plant community dynamics^5^.

The European wood wasp *Sirex noctilio* Fabricius, is a devastating killer of pine trees in the Southern hemisphere. It was first discovered in New Zealand^6^ outside its native range of Europe, North Africa, and the Middle East. Over the twentieth century, it has invaded exotic pine plantations in New Zealand, Australia, South America and South Africa successively^7^, into the northern hemisphere in the northeastern United States and southeast Canada in 2004 and 2005, respectively, and in South America^7^. Interestingly, as a secondary pest of pine species, this insect is not considered a pest in Europe^6,8^, but in several countries of the southern hemisphere and North America, the woodwasps has attracted considerable attention due to its high invasive ability and ability to kill a variety of pine species^9,10,11^. In August 2013, the woodwasps were first detected as a pest of Mongolian pine in the Duerbote Mongolian Autonomous County, Heilongjiang Province, China. To date, Mongolian pine plantations are considered to be in danger of the infestation by the woodwasp over 22 cities in northeast China^12,13^. *Sirex noctilio* damages pine trees by depositing an obligate symbiotic fungus, *Amylostereum areolatum* (Fr.) Boidin and a phytotoxic mucus in the trees during oviposition. The toxic mucus affects tree defenses and assists the colonization of fungus in the host. The symbiotic fungus acts as an external gut of the woodwasp larvae for the digestion of recalcitrant lignocellulosic compounds^14,15^. Thus, insects, toxins, and fungi act together to damage host trees.

Mongolian pine tree decline is commonly considered as a multifactorial disease, in which many interacting abiotic and biotic factors such as drought, frost, insect pests and pathogens are involved. Among the biotic factors involved in the onset of Mongolian pine tree decline, pathogenic fungi play a primary role. So far, more than 20 fungi diseases of Mongolian pine trees have been reported^4,5^. In particular, many independent surveys have demonstrated the involvement of some leaf blight agents such as *Lophodermium seditiosum* Minter Stalay and Millar^16^, *Coleosporium phellodendri* Kom, *Lophodermella sulcigenaa* (Link) Tubeuf^17^, *Septoria pinipumilae* Sawada^18^, and trunk parts agents such as *Cronartium quercuum*^19^ (Berk.) Miyabe ex Shirai and *Cronartium flaccidum* (Alb. et Schw) Winter^20^, and root rot agents such as *Rhizoctonia solani* Kühn^21^, in the Mongolian pine decline processes. However, in recent years, it is shown that the colonization of some important invasive pests may contribute to accelerating the Mongolian pine tree decline. It is unknown whether the decline of *P. sylvestris* var*. mongolica* forest invaded by *S.* *noctilio* is related to the fungal communities in host trees.

Previous studies have shown that the declining trees were preferentially infested by the woodwasps, however, when the population density was high, they also infested the healthy trees^10^. A number of pathogenic fungi have been recognized as having a prominent role in Mongolian pine tree decline and mortality^5^. The most common method of the investigation is to cut down the declining pine trees to control the woodwasp and has achieved great results. One drawback of many investigations, however, was from single, separate disciplines (e.g., climatologists, plant pathologists, entomologists, etc.), and led to broach only one possible cause at a time, without a comprehensive, holistic approach to the problem^22^. The result was in many instances a disjointed and often incomplete, which made it impossible to determine the real causes of tree declines.

*Sphaeropsis sapinea* is an important latent pathogen of *Pinus* spp. and widely distributed in *P. radiata* plantations in northern Spain. It was recognized as the most widespread necrotrophic ascomycete pathogen responsible for dramatic losses of pine trees across the continents^23,24^. *Sphaeropsis sapinea* has emerged as an aggressive fungal pathogen all over the world and could directly invade the young shoots of pine trees^25–27^. In the greenhouse experiment, Stanosz and Flowers proved that *S. sapinea* strains isolated from healthy and diseased pine tissues had high pathogenic potential^28,29^. Usually, numerous pycnidia of *S. sapinea* are present in forest stands occurring on twigs, needles branches and stems of pine trees. A high infection rate may pose a high risk to forests when there are disease-triggering factors, e.g., hail or insect feeding or extreme weather conditions such as heat and drought, as in the years 2018 und 2019 in German^24^.

The frequency of pine shoot blight on pine trees has significantly increased over the past decades in southeast China, especially in mature pine forests^5^, and the loss caused by *S. sapinea* is no less than that by *Bursaphelenchus xylophilus* (the most serious invasive species of conifer trees in China). In northeast China, the infection with *S. sapinea* in Mongolian pine trees was also reported^30^. However, the reasons that cause the decline of the Mongolian pine forests invaded by *S. noctilio* are unknown.

In this study, we hypothesize that the fungal disease acts as the initial reason for the decline of the Mongolian pine forest in northeast China, the woodwasps attack the infected and stressed trees and accelerate the decline. We investigated the fungal species occurring in three tree samples: *S. noctilio* infested trees, healthy uninfested trees, unhealthy uninfested. We analyzed the relationship between the decline of pine trees and the occurrence of pathogenic fungi and the woodwasp infestation. In addition, we also studied the pathogenicity of *S. sapinea* to healthy *P. sylvestris* var. *mongolica* trees*.* The findings obtained in this study allow us to characterize, for the first time, the relationship between the decline of Mongolian pine trees and fungal communities and invasive pest in northeast China pine plantations.

## Results

### Number of *S. noctilio* and other borers

**Figure 1:**
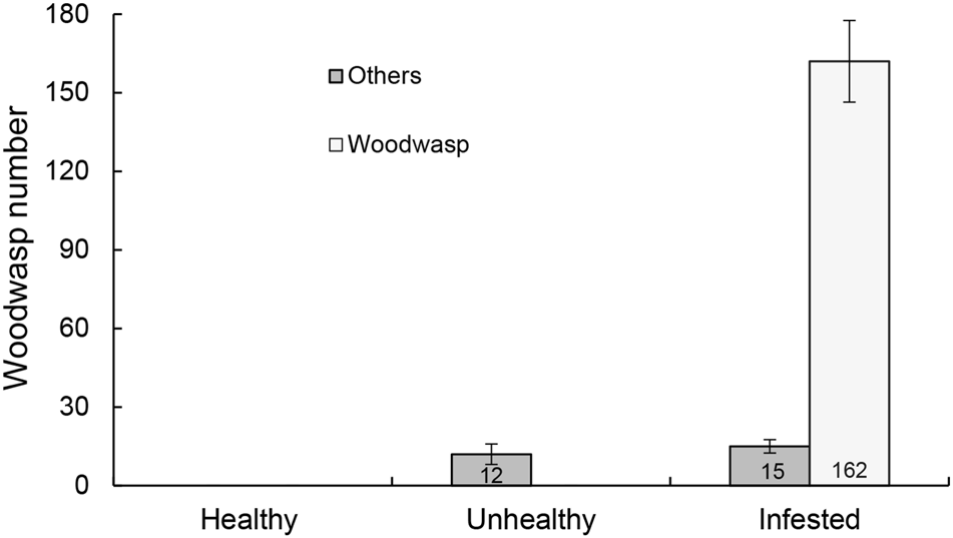
The number of *S. noctilio* and other species of woodboring insects in different sampled trees. Bars and brackets are means and standard error, respectively. Numbers inside the bottoms of the bars are the number of *S. noctilio* or other borers. *Healthy* Healthy uninfested trees, *Unhealthy* Unhealthy uninfested trees, *Infested* *S. noctilio* infested trees.

In total 162 *S. noctilio* individuals, including 141 adults and 21 larvae (16 dead and 5 alive) from tree bolts, were observed from the infested trees, but no *S. noctilio* individual was found in healthy uninfested and unhealthy uninfested trees (Fig. 1).

Apart from the woodwasp which specifically attacks *P. sylvestris* var. *mongolica*, other wood-boring pests (all of them were bark beetles) were eventually found in sampled trees. The number of bark beetles collected was 12 and 15 in unhealthy uninfested and infested trees, respectively (Fig. 1). No insects were found in healthy uninfested trees. Native bark beetles (*Ips sexdentatus*) were collected not only in *S. noctilio* infested trees, but also in unhealthy uninfested trees. The number of *S. noctilio* had a significant positive correlation with the number of native bark beetles (Table 1).

**Table 1:** Phi (φ) coefficients for within-tree associations for *S. sapinea* and Boring pests.

	*Sirex noctilio*	Bark beetle
*Sphaeropsis sapinea*	0.189 *	0.164 *
*Sirex noctilio*	–	0.232 **
Bark beetle	–	–

*P < 0.05; **P < 0.01; ***P < 0.001.

### Structure of fungal communities from three tree samples

**Figure 2:**
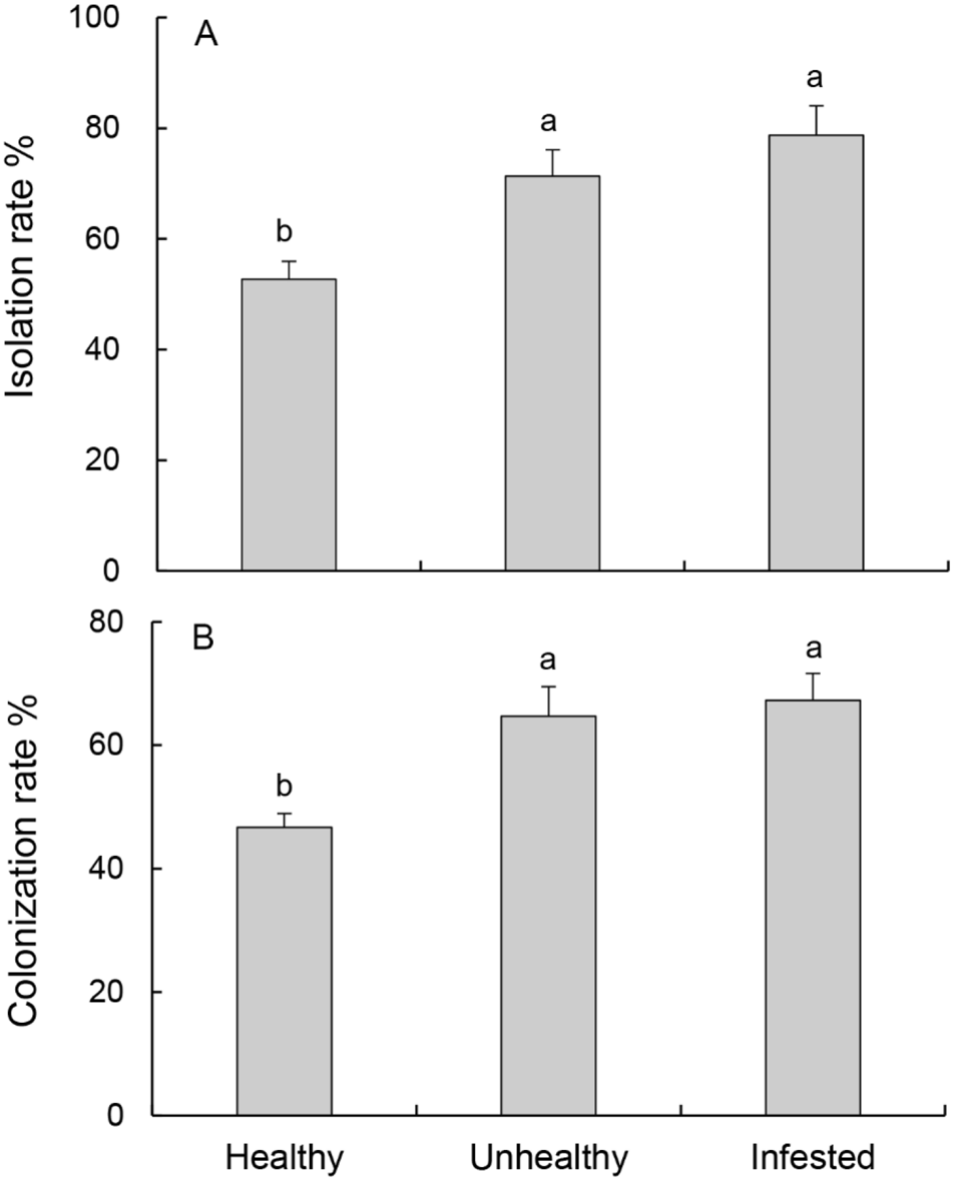
The rates of isolation (**A**) and colonization (**B**) of endophytic fungi from three tree samples. Bars and brackets are means and standard error, respectively. Different lowercase letters indicate a significant difference between the isolation rates or colonization rates in different tree samples at p < 0.05.

A total of 450 wood fragments of healthy uninfested, unhealthy uninfested and *S. noctilio* infested trees were evaluated for the occurrence of endophytic fungi. The colonization rates (CR) and isolation rates (IR) of endophytic fungi between three samples were significantly different (CR: F = 10.64, df = 2, p < 0.05; IR: F = 8.7, df = 2, p < 0.01) (Fig. 2). There was no significant difference in the CRs and IRs between infested and unhealthy uninfested trees (CR: F = 0.26, df = 1, p > 0.05; IR: F = 1.04, df = 1, p > 0.05). In addition, the CRs and IRs of pathogenic fungi in *S. noctilio-*infested and unhealthy trees were significantly higher than that of healthy trees (CR: F = 11.26, p < 0.05; IR: F = 7.52, p < 0.01) (Supporting information Figure S1).

The isolated endophytic fungi (304 in total) were assigned to 26 species within 21 genera based on their ITS sequence data and morphological features (Table 2). Among the 21 genera, 19 genera (24 species) were within the phylum Ascomycota, and 2 genera (2 species) were within the phylum Basidiomycota. Among the 26 species (there were overlapping fungi species in different samples), 11 endophytic fungi species were isolated from healthy uninfested trees, including *Chaetomium globosum* (26.6%), *Sphaeropsis sapinea* (16.5%), *Alternaria alternata* (12.6%) and *Trichoderma atroviride* (11.4%). From unhealthy uninfested trees, 13 fungal species were isolated, and the most frequent fungal isolates were *S. sapinea* (47.7%) and *T. atroviride* (14%) (Table 2). From *S. noctilio* infested trees, 16 fungi species were isolated, and the dominant fungi species were *S. sapinea* (37.3%), *Ophiostoma minus* (22%) and *T. atroviride* (11.9%) (Table 2).

**Table 2:** Colonization number and significance for forestry of fungal endophytes isolates obtained from three tree samples.

Fungal taxa	Accession number		Closest species (Accession No.)	Similarity	Health uninfested	Unhealthy uninfested	Infested	Assessment of the significance for forestry^a^
				(%)				
*Aspergillus tubigensis*	MT994717		*Aspergillus tubigensis* (GU595290)	99	2		1	_
*Aspergillus niger*	MT994716		*Aspergillus niger* (KP940593)	100	6	5	1	Endophyte^54,55^
*Alternaria alternata*	MT994718		*Alternaria alternata* (KJ173524)	99	9	7	3	Generalist^62^
*Amylostereum areolatum*	MT994715		*Amylostereum areolatum* (KC865582)	100			1	Symbiotic fungi of wasps, saprophyte^6^
*Bionectria ochroleuca*	MT994719		*Bionectria ochroleuca* (HM037945)	99			2	Biocontrol^63^
Botrytis cinerea*	MT994722		*Botrytis cinereal* (MH860108)	100		2		Pathogen^16,64^
*Chaetomium globosum*	MT994720		*Chaetomium globosum* (KM268644)	99	21			Biocontrol, typical endophyte of Mongolian Pine^51,64^
*Epicoccum nigrum*	MT994725		*Epicoccum nigrum* (AF455403)	99			3	Generalist^65^
Fusarium tricinctum*	MT994723		*Fusarium tricinctum* (EF611089)	100	8	3		Endophyte, potential pathogen, potential pathogen^31,54^
Fusarium chlamydosporum*	MT994724		*Fusarium chlamydosporum* (MG857338)	99			1	Potential pathogen^54^
*Fusarium solani* complex*	MT994721		*Fusarium solani* (EU719658)	99			7	Pathogen, saprophyte^66^
*Gliomastix* sp.	MT994727		*Gliomastix polychrome* (AB540566)	97	1			_
Leptographium lundbergii*	MT994733		*Leptographium lundbergii* (DQ062031)	99		5	8	Blue stain of wood, pathogen^67^
*Nectria haematococca*	MT994726		*Nectria haematococca* (MH729023)	99		1		_
Ophiostoma floccosum*	MT994728		*Ophiostoma floccosum* (KF854000)	99			1	Pathogen^71^
Ophiostoma minus*	MT994729		*Ophiostoma minus* (GU134172)	100		11	26	Pathogen, associated fungi of bark beetles, blue stain of wood^48^
*Penicillium glabrum*	MT994735		*Penicillium glabrum* (HG326279)	99	7			_
*Peyronellaea* sp.	MT994734		*Peyronellaea* sp. (KF293765)	99	2		-	_
Phoma multriostrata*	MT994731		*Phoma multriostrata* (EF585395)	100		2	1	Pathogen^31^
*Pestalotiopsis* sp.	MT994730		*Pestalotionpsis lespedezae* (FJ467379)	99		1		_
Sphaeropsis sapinea*	MT994737		*Sphaeropsis sapinea* (HM467670)	99	13	51	44	Typical pathogen of pine shoot blight, saprophyte^25,27,28^
*Schizophyllum commune*	MT994736		*Schizophyllum commune* (MK910781)	99	1			Saprophyte^68^
Sydowia polyspora*	MT994732		*Sydowia polyspora* (KU319069)	99		1		Typical endophyte of *P. sylvestris* twigs, Potential pathogen^69^
Truncatella angustata*	MT994738		*Truncatella angustata* (KU319069)	99		3	1	Saprophyte, weakness pathogen^70^
*Trichoderma atroviride*	MT994739		*Trichoderma atroviride* (HM037962)	100	9	15	14	Endophyte^32^
*Trichoderma viride*	MT994740		*Trichoderma viride* (HM037962)	100			4	Endophyte^32^

*Plant pathogen fungi.

^a^The numbers in the column that assessment of the significance for forestry were references number.

**Figure 3:**
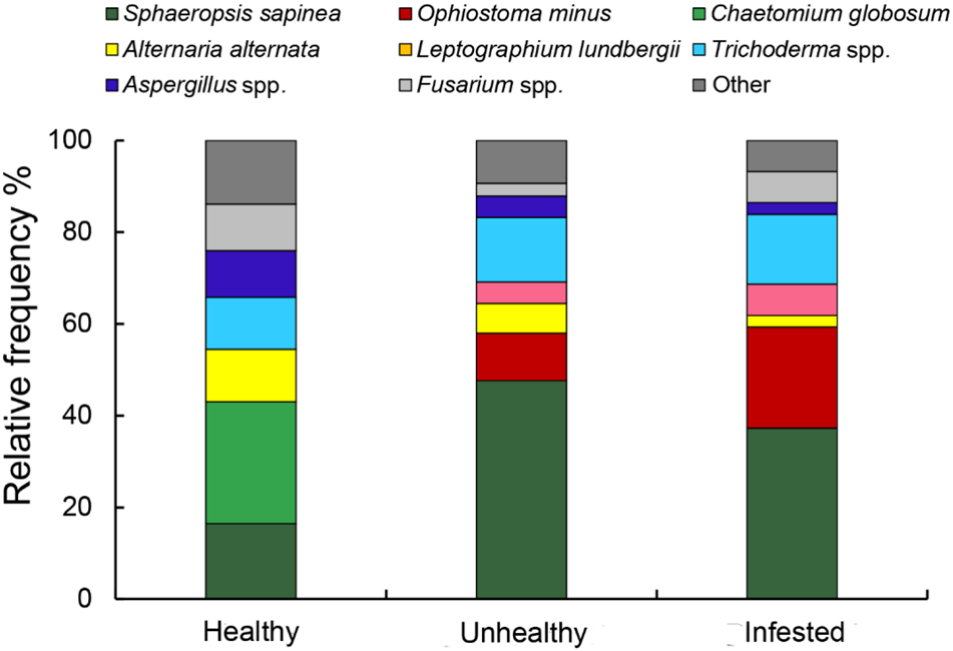
Composition of the most frequently isolated fungi from the different tree samples. The eight most frequently cultivated species (genera) were selected and the prevalence (%) of each species (genus) was determined per tree samples.

Top-eight most prevalent fungi species (genera) accounted for 90% of all the isolates, ranging from 86.1 to 92.3% (Fig. 3). The relative frequency of *S. sapinea* in healthy uninfested trees was lower than those of *S. noctilio* infested and unhealthy uninfested trees. The relative frequency of *Trichoderma* spp. was slightly higher in *S. noctilio* infested (15.3%) and unhealthy uninfested trees (14%) than that of healthy uninfested trees (11.4%), and *Aspergillus* spp. and *Fusarium* spp. in healthy uninfested trees were higher than the other two tree samples.

**Figure 4:**
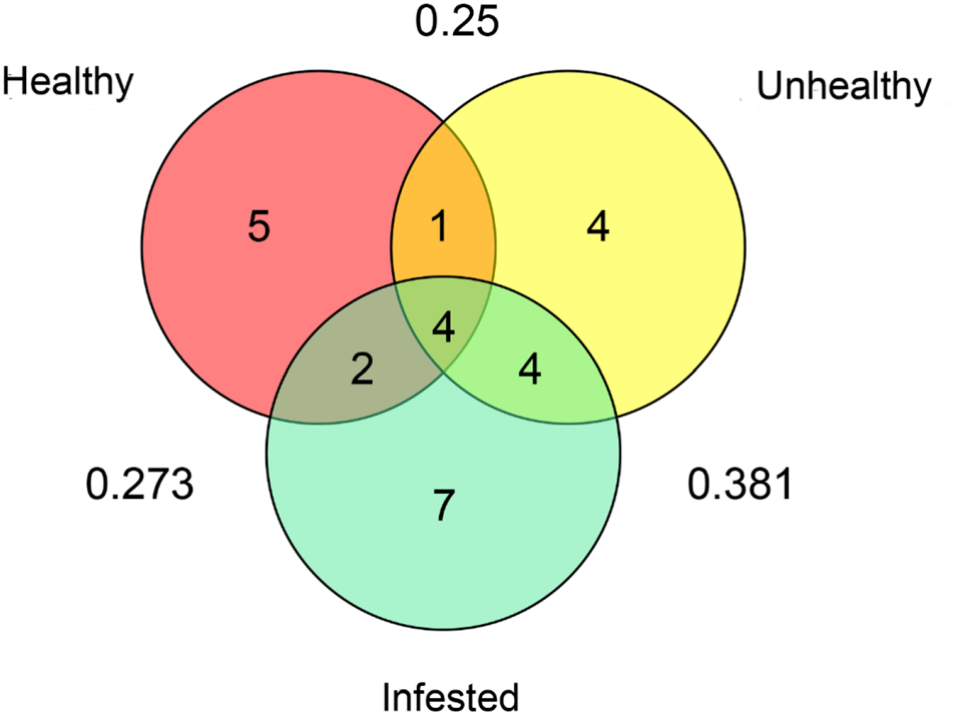
Venn diagram illustrating the unique and shared fungal taxa among healthy trees (red), unhealthy trees (yellow), and *Sirex noctilio* infested trees (green). Outside numbers are the Jaccard similarity coefficient.

Four fungal species, namely *Aspergillus niger*, *A. alternata*, *S. sapinea*, and *T. atroviride* were isolated from three tree samples, and common in three tree samples accounting for 15.3% of all the species. The highest overlap (Jc = 0.381) was observed for the fungal communities between *S. noctilio* infested and unhealthy uninfested trees (Fig. 4). Some fungal species only existed in a single tree sample (healthy uninfested: 5 species; unhealthy uninfested: 4 species; infested: 7 species). The species *Leptographium lundbergii* and *O. minus* were isolated from *S. noctilio* infested and unhealthy uninfested trees, whereas *C. globosum* was only species isolated from healthy uninfested trees (Table 2; Fig. 3).

A total of 11 pathogenic species were identified from three tree samples, including 2 pathogenic species from healthy uninfested trees, 8 pathogenic species from unhealthy uninfested trees and 8 pathogenic species from *S. noctilio* infested trees (Table 2; Supporting information Figure S1). The pathogenic fungi richness was four times higher in infested and unhealthy uninfested trees than in healthy uninfested trees. Some pathogenic fungi found in unhealthy trees were also isolated from healthy trees uninfested by *S. noctilio.* For example, *S. sapinea* (pathogen of pine shoot blight) was isolated from all three samples and the isolation rate was significantly higher compared to other fungi. *S. sapinea* exhibited positive interspecific associations with the within-tree colonization *S. noctilio* and bark beetle (Table 1).

### Diversities of the fungal community

The diversity indexes of endophytic fungal communities showed significant differences among the three tree samples (Shannon diversity index: F = 6.72, df = 2, p < 0.05; Simpson dominance index: F = 43.47, df = 2, p < 0.05; Richness index: F = 21.25, df = 2, p < 0.05). The Shannon diversity index was higher, and the Richness index was lower for the fungal community from healthy uninfested trees than those from infested and unhealthy uninfested trees (Table 3). The Richness index was the highest in *S. noctilio* infested trees compared to other two tree samples. The Simpson dominance indexes of infested and unhealthy uninfested trees were higher than that of healthy uninfested trees, demonstrating that fungal communities under these two conditions had a high concentration compared to healthy trees. In addition, the Simpson dominance index was slightly higher in unhealthy uninfested trees than that in the infested trees community (F = 1.78, df = 1, p > 0.05).

**Table 3:** Diversity indices of fungal communities from three tree samples.

Index	Healthy uninfested	Unhealthy uninfested	Infested
Shannon diversity index	2.123 ± 0.14a	1.807 ± 0.08b	1.958 ± 0.11ab
Simpson dominance index	0.145 ± 0.05b	0.269 ± 0.07a	0.213 ± 0.03a
Richness index	2.517 ± 0.08b	2.568 ± 0.12b	3.144 ± 0.15a

The data were analyzed by one-way ANOVA followed by HSD test. The results are expressed as the mean ± SD. The results followed by different letters are significantly different according to the HSD test (p < 0.05).

### Infection ability of *S. sapinea* to healthy *P. sylvestris* var. *mongolica* trees

The incidence rates of *S. sapinea* infection to the needles of healthy *P. sylvestris* var*. mongolica* trees were significantly different in the two treatment groups (wounded + spore and nonwounded + spore) compared with that in the two negative control groups (wounded + water and nonwounded + water) (F = 318.74, df = 3, p < 0.01) (Table 4). *S. sapinea* showed strong pathogenicity to the wounded *P. sylvestris* var*. mongolica* needles (incidence, 83.22%), which eventually caused the needles to wither. However, *S. sapinea* could also penetrate *P. sylvestris* var. *mongolica* without wounding but with a lower incidence of 38.72% (F = 122.99, df = 1, p < 0.01). In addition, pathogenic fungi re-isolated from diseased needles were the same as in the inoculum used for the healthy needles previously (Table 4). In the two negative control groups, the needles of healthy *P. sylvestris* var. *mongolica* trees could hardly be infected, regardless of whether they were wounded or not.

**Table 4:** Pathogenicity of *S. sapinea* to healthy *P. sylvestris* var. *mongolica.*

Inoculation treatments	Number of inoculation needles	Number of diseased needles	Incidence (%)	Re-isolated from diseased needles	Same as inoculated fungus
Wounded + spore	304	253	83.22 ± 10.45 a	20	20
Nonwounded + spore	359	139	38.72 ± 8.14 b	20	20
Wounded + water	267	6	2.25 ± 4.46 c	–	–
Nonwounded + water	380	0	0.00 ± 0.00 c	–	–

Incidence followed by different letters were significantly different according to the Tukey’s HSD test (p < 0.05).

## Discussion

Recently, the woodwasps have been found in declining Mongolian pine woodlands in northeast China^12^. The current study revealed a positive association between *S. noctilio* and native bark beetles (*Ips sexdentatus*) as reported previously (Table 1)^31^. However, the population number of the bark beetles was lower and not considered as a pest in northeast China over the past several years^30^. The current study also found 162 woodwasps only from *P. sylvestris* var. *mongolica* trees with signs of wasp egg laying (Fig. 1) and in unhealthy and *S. noctilio* infested *P. sylvestris* var. *mongolica* trees, as reported previously that this insect preferred to damage declining pine species^32–34^. However, the Mongolian pine woodlands had been declining before the invasion of *S. noctilio* in northeast China. This declining may be due to the fungal community in the Mongolian pine woodlands, which provides convenience for the invasion of *S. noctilio*^12,30^*.* The woodwasps attack stressed trees, particularly disease-stressed ones, which are their preferred hosts^35^. This accelerates the decline and even death of Mongolian pine trees.

The association of Mongolian pine decline with fungal communities has been shown previously^4^. In this study, a total of 26 fungal species was isolated from three tree samples. Pathogenic fungi richness was four times higher in the *S. noctilio* infested and unhealthy uninfested trees than that in the healthy uninfested trees (Table 2; Supporting information Figure S1). Some of common pathogens of pine needles constitute a danger to weakened pine stands^36^. *Ophiostoma minus* was the second most common fungus in this study. It was reported that the woods colonized by *O. minus* dries more quickly^37^. *Leptographium lundbergii* and *O. minus* are considered as blue stain fungus of different pine trees worldwide, which is introduced to pine trees by bark beetles^38,39^. In contrast, *C. globosum* was the most frequent fungal isolates only found in the healthy uninfested trees (Table 2). It is a biocontrol fungus as it produces various secondary metabolites and enzymes capable of inhibiting the mycelia growth of pathogenic fungi^40,41^. Recent research showed that *C. globosum* completely inhibited the mycelial growth of *Amylostereum areolatum*^42^.

*Amylostereum areolatum* showed up only in one sample in *S. noctilio* infected trees although the woodwasp attack on trees was widespread in this study. The growth rate of *A. areolatum* is low and the ability of occupying the resources and niche is weaker than many other endophytic fungi present in the pine ecosystems. Previous research found that some endophytic fungi can inhibit the mycelial growth of *A. areolatum* and destroy the mutual symbiotic relationship between *S. noctilio* and *A. areolatum* in host trees^42,43^. In addition, in this experiment, the xylem tissue of *P. sylvestris* var. *mongolica* trees was selected randomly, and the spawning site of the woodwasps was not selected specifically, so the number of isolated symbiotic fungi was very small.

Previous studies have found that the species of endophytic fungi are closely related to the health level of trees^43^. In this study, *Trichoderma*, *Aspergillus* were the dominant genera of endophytes (Table 2) as reported in different host plants^44–46^. The Richness index showed that endophytic fungi species in the *S. noctilio* infested trees were the highest. The CR and IR values of endophytic fungi of the healthy uninfested trees was the lowest compared with the values of the *S. noctilio* infested and unhealthy uninfested trees (Table 2; Fig. 2). The highest similarity (0.38) was observed for the fungal communities between the *S. noctilio* infested and unhealthy uninfested trees (Fig. 4). The results show that the fungal community structure is greatly affected by tree health conditions^43^.

On the other hand, the invasion of the woodwasps accelerated host decay and promoted the colonization of saprophyte, such as *Fusarium solani*^40,47^. For example, the symbiotic fungus was only isolated from the *S. noctilio* infested trees in this study. Furthermore, no significant differences were observed in the CR or IR values between the unhealthy uninfested and *S. noctilio* infested trees. The primary endophytic fungal species from unhealthy uninfested and *S. noctilio* infested trees were also similar. The results of Simpson dominance index showed that fungal communities had a high concentration in unhealthy uninfested trees compared to that in other two tree samples (Table 3).

In this study, *S. sapinea* was the most abundant species obtained from tree trunks of *S. noctilio* infested (37.3%) and unhealthy uninfested (47.7%) trees (Table 2) and showed a very strong pathogenicity and could penetrate *P. sylvestris* var. *mongolica* without wounding (Table 4). *S. sapinea* is the causal fungal agent of Diplodia tip blight disease to the coniferous trees of relevance to forestry in the world (Supporting information Table S1). The severity of pathogenicity, the length of incubation period and propagation period of the fungi are related to the host tree vigor, tissue maturity and environmental conditions^44^. Palmer reported that *S. sapinea* strains from China could invade pine trees without wounding, while those from the United States could not^48^. However, Blodgett found that *S. sapinea* strains from the United States could also invade pine trees without wounding, but the incidence was low^49^. The occurrence of *S. sapinea* in healthy pine trees of this study, measured in frequency of colonization, is higher than in other studies like by Zhou^50^ and Maresi^51^. In addition, the occurrence of *S. sapinea* had positive associations with both *S. noctilio* and bark beetle (Table 1), which could be driven by the attraction of the unhealthy trees infested by *S. sapinea* to the woodwasps to oviposit during host selection.

In our opinion, *Pinus sylvestris* var. *mongolica* forests in northeast China are being damaged by *S. sapinea* and other fungi, and these fungal diseases was getting worse year by year, causing the trees to decline^44^. Their infection may promote convenient conditions for the successful colonization of *S. noctilio*. Therefore, we considered the decline of Mongolian pine forests could be the result of the combinatory effects of *S. noctilio* and plant pathogenic fungi.

## Materials and methods

### Study sites and wood sample collection

The research site was in the Jun De Forest Farm (130° 17′ 47′′ E, 47° 12′ 11′′ N) in Hei longjiang Province, China, which is comprised primarily of 25–30 years old *P. sylvestris* var. *mongolica*, *P. koraiensis*, *Picea koraiensis*, and *Larix gmelinii* plantations. The site was characterized by a cold climate with an average annual temperature of 3.7℃ and average annual precipitation of 600 ~ 650 mm. The Mongolian pine trees selected in this study came from the sample plot of pure *P. sylvestris* var. *mongolica* forests (with an area of 2 hectares) previously investigated (unpublished data) and has not been thinned since planting. Some trees showed signs of decline and had been damaged by the wasp *S. noctilio* as previously reported^13,52^. In April 2018, fifteen trees were randomly chosen from the pure *P. sylvestris* var. *mongolica* plantation and listed in Table 1, including three groups: 5 *S. noctilio* infested trees, 5 healthy uninfested trees and 5 unhealthy uninfested trees. The *S. noctilio* infestation of Mongolian pines was identified by typical oviposition symptoms (i.e., resin beads formed from each ovipositor insertion). The distance between individual trees was at least 10 m. In fact, the distance between the uninfested healthy trees and *S. noctilio* infested trees was 10 m, and the distance between other trees exceeded 30 m (Supporting information Figure S2).

Fresh wood samples were collected from tree trunk segments of 2 m above ground^44^ (Table 5). Briefly, a trunk disk (10 cm-thick cross-section) was cut off from the segment. A bark layer more than 1 cm thick was removed from the disk using a sterile knife. Next, a sample block (10 × 10 × 5 cm^3^) was removed from each disk and sealed in a sterile vacuum bag. All sample blocks were transferred to the laboratory at Gansu Agriculture University and stored at 4 °C (up to 2 weeks) until further analyses.

**Table 5:** Selection of tree samples.

Tree samples	Diameter (cm)^a^	Height (m)	Dead branches and leaves (%)	Infestation^b^	Moisture content (%)
Healthy uninfested	16.91 ± 1.42a	7.71 ± 1.11a	6.20 ± 3.8b	Without	72.13 ± 4.11a
Unhealthy uninfested	16.78 ± 1.27a	8.02 ± 1.22a	50.00 ± 5.7a	Without	61.97 ± 3.25b
Infested	16.72 ± 1.38a	7.83 ± 0.65a	52.00 ± 6.51a	Woodwasp	62.91 ± 6.22b

^a^Diameter: The diameter of 2 m above ground from each tree.

^b^Infestation: Whether insect infected the tree samples before sampling.

### Collection of pests from *Sirex* infested trees

Fifteen Mongolian pine trees were cut into 1 m-long billets after the wood sample collection, excluding the bottom 1 m section, with a minimum 10 cm diameter. After sealing the cut ends with wax, sample logs were taken to the quarantine laboratory in Gansu Agriculture University. These logs from visually identified tree samples (healthy uninfested, unhealthy uninfested, infested) were individually placed in mesh cages in the rearing facility and maintained at 27 ± 3 °C temperature and 65 ± 5% relative humidity (RH) until the adult *S. noctilio* emerged. The adult numbers of *S. noctilio* and other pests were counted from the tree samples. In addition, the wood sample were split to count the number of *S. noctilio* larvae after the adult emergence.

### Isolation and storage of endophytic fungi

Endophytic fungi were isolated from the sample blocks using a surface sterilization method^53^. Briefly, each sample block was cut with a sterile pruner into 30 fragments (size: 4 ~ 5 mm^3^). The fragments were surface sterilized by dipping in a series of solutions (70% ethanol for 1 min, 12% sodium hypochlorite for 30 s, and 70% ethanol for 1 min). They were then washed three times in sterile distilled water. Five surface-sterilized fragments were placed in a petri dish (90 mm) with potato dextrose agar (PDA: 200 g potato, 20 g glucose, 15 g agar, and 1L distilled water) supplemented with 100 μg/mL ampicillin and 50 μg/mL chloramphenicol. All fragments were incubated at 25 ± 1 °C and 70 ± 5% RH for 1 ~ 4 weeks or until the emergence of fungal mycelium. Agar cubes (ca. 1 mm^2^) were removed aseptically from the edge of fungal colonies and transferred to fresh PDA plates. Each fungal colony was transferred at least three times until a well-defined uniform culture was obtained. Purified fungal isolates were sub-cultured with half-strength PDA in 60-mm Petri dishes and kept on the laboratory bench at about 20 ~ 25 °C, where they received indirect sunlight to enhance sporulation. The fungal isolates were initially grouped as representative isolates and classified by their macro- and micro-morphological features, such as colony appearance, size, and shape of spores with species descriptions available in the literature^54^.

The fungal cultures were generated on PDA slants in centrifuge tubes and stored under sterile mineral oil at 4 °C. For long-term preservation, the representative isolates of each taxon were transferred to 20% glycerol in ultra-clean distilled water (v/v) and stored at − 80 °C.

### Molecular identification of isolates

For the determination at the species level, the representative isolates of each taxon identified by the morphological features above were grown on PDA and incubated at 25℃ in the dark using InstaGene Matrix (BioRad Laboratories, Hercules, CA, USA). Genomic DNAs were extracted from 5-day-old cultures. The primers ITS1 and ITS4^55^ were used to amplify the internal transcribed spacer (*ITS*) regions by PCR. The PCR reactions were carried out in a volume of 25 μL using 23 μL Golden Medal MIX (Thermo Scientific, USA), 1 μL of each primer (10umol/L), and 1 μL template DNA (50 μg/mL). The PCR amplification was conducted using the following conditions: an initial denaturation step of 98 °C for 2 min; followed by 30 cycles of denaturation at 98 °C for 10 s, annealing at 50 °C for 15 s, and polymerization at 72 °C for 15 s; and then a final extension step of 5 min at 72 °C.

The PCR products were separated by electrophoresis on 1% (w/v) agarose gels, stained with ethidium bromide for visual examination, and purified using the agarose gel DNA extraction kit (Takara, Japan) and sequenced at Qinke Biotech (Beijing, China). The sequences were submitted for BLAST search in the GenBank (http://blast.ncbi.nlm.nih.gov/Blast.cgi). The representative isolates were assigned to a species when their sequences were at least 99% identical to the sequence of a known species. Besides, morphological features of the representative isolates were also used an important role to confirm the classification by the DNA sequence comparison. The following morphological features were evaluated: mycelium shape, mycelium surface texture, colony color, production of pigments and their diffusion in the medium, spore production, and mycelium growth rate on the PDA plates.

### The pathogenicity test of *Sphaeropsis sapinea *to *P. sylvestris* var. *mongolica*

In this experiment, the *S. sapinea* (synonym: *Diplodia pinea*, pine shoot blight) that were isolated from Mongolian pine forest in the previous step was selected for pathogenicity test with *P. sylvestris* var. *mongolica*. Before inoculation, *S. sapinea* was cultured intermittently under black light (100 ~ 150 lx) for 14 h and in the dark for 10 h on PDA + M medium (PDA + sterilized powder of Mongolian pine needles) for 20 days at 25 ± 1 °C and 70 ± 5% RH to induce fungal sporulation^37^, and then the spores were washed with sterile water and 50 spores were collected under a low power microscope (Wincom, China) to make into fungus suspension. Then, the inoculation experiment was conducted on the needles of 3-year-old healthy seedlings of *P. sylvestris* var. *mongolica* in the laboratory^30^. First, the needles of *P. sylvestris* var. *mongolica* seedlings were stabbed with a sterile knife at the base of the needles, with one wound per needle. The uninjured needles were used as a control treatment. Then, the fungus suspension, prepared as above, was smeared on the stabbed needles of *P. sylvestris* var. *mongolica* with a brush and bound with self-adhesive plastic film for 10 days. The uninjured and stabbed needles smeared with sterile water served as negative controls. In this experiment, the fungus was inoculated twice (once more after 10 days). Ten independents healthy seedlings of *P. sylvestris* var. *mongolica* were used for each of the four treatments (stabbed and uninjured needles smeared with fungus spores and with water) with 19 ~ 54 needles each seedling. The incidence rates of *S. sapinea* were investigated after 3 months post-inoculation. After the incidence rate of needle infections was determined, *S. sapinea* was re-isolated from 20 diseased needles randomly selected from each treatment group.

### Data analysis

The colonization rate (CR) was calculated as the number of tree fragments from which one or more endophytic fungi were isolated, divided by the total number of incubated trees fragments^56^. The isolation rate (IR) was defined as the number of endophytic fungi isolated, divided by the total number of tree fragments incubated^57^. The incidence rate was calculated as the number of diseased needles, divided by the total number of inoculation needles. The CR and IR of endophytic fungi and the incidence of *S. sapinea* to healthy *P. sylvestris* var. *mongolica* were analyzed using one-way ANOVA. The differences between mean values were evaluated using Tukey’s honestly significant differences (HSD) test. Pearson’s chi-square test was applied to analyze the differences between pathogenic fungi and other fungi (remaining fungi except for pathogenic fungi) from each tree sample. We analyzed within-tree correlations of presence/absence of *S. sapinea* and pests using phi (φ) coefficients. The statistical analyses were performed using the IBM SPSS Statistics version 23.0 (Chicago, IL, USA). The relative frequency of the common fungi isolated from each tree sample was examined using the range diversity analysis^58,59^.

The diversity of endophytic fungi isolated from each tree sample was evaluated using the Shannon–Weiner Index (*H*′), Simpson dominance index (*D*), and Margalef richness index (*R*)^60^.

$$ H^{\prime} = - \sum (Pi \times \ln Pi). $$

$$ D = 1 - \sum Pi^{2} $$

$$ R = (S - 1)/\ln N $$

$$ Pi = Ni/N $$

where *N* is the total number of individuals; *Ni* refers to the number of individuals; and *S* indicates the total number of species. In addition, the similarity of fungal communities was evaluated using the Jaccard similarity coefficient (Jc)^61^. The similarities in fungal taxonomic richness between communities were summarized in Venn diagrams using GeneVenn software (http://genevenn.sourceforge.net/).

### Informed consent

All experimental protocols were approved by Biocontrol Engineering Laboratory of Crop Diseases and Pests of Gansu Province, Gansu Agricultural University, Lanzhou, China. All the methods were carried out in accordance with the relevant guidelines and regulations.

## Supplementary Information

Supplementary Information.

## Data Availability

We declare that all the date in this study were available.
