# Supplementary material for: Mongolian pine forest decline by the combinatory effect of European woodwasp and plant pathogenic fungi
Source: Sci Rep. 2021 Oct 4;11:19643. doi: 10.1038/s41598-021-98795-y (PMC8490441; doi:10.1038/s41598-021-98795-y)
Supplement: Supplementary file 1 — Supplementary Information. [file 41598_2021_98795_MOESM1_ESM.docx]

Figure S1


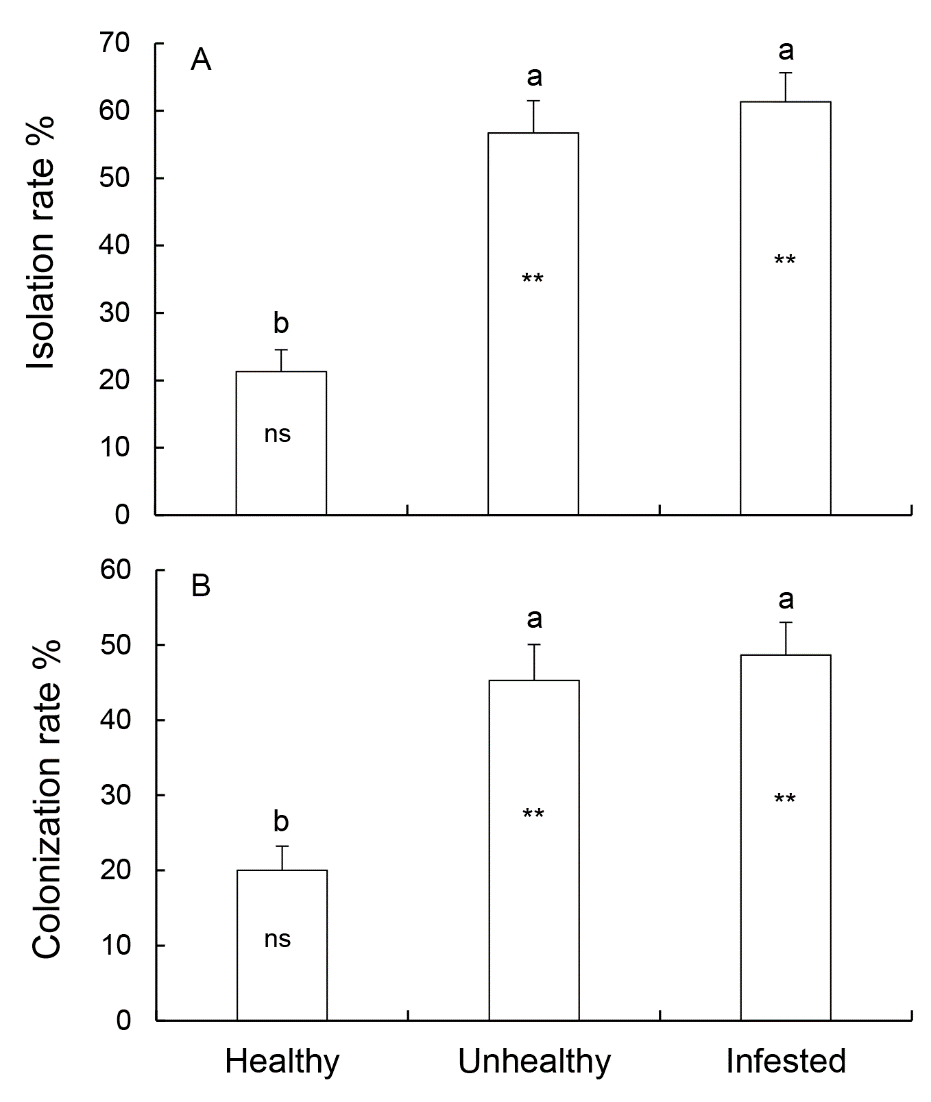


**Figure S1.** The rates of isolation (**A**) and colonization (**B**) of pathogenic fungi from three tree samples. Lowercase letters indicate a significant difference between the isolation rates or colonization rates in different tree samples at *P* < 0.05. The differences between pathogenic fungi and other fungi (remaining except for pathogenic fungi) from each tree sample was analyzed by Pearson’s chi-square test, ns: *P* ≥ 0.05; *: *P <* 0.05; **: *P <* 0.01.

Figure S2


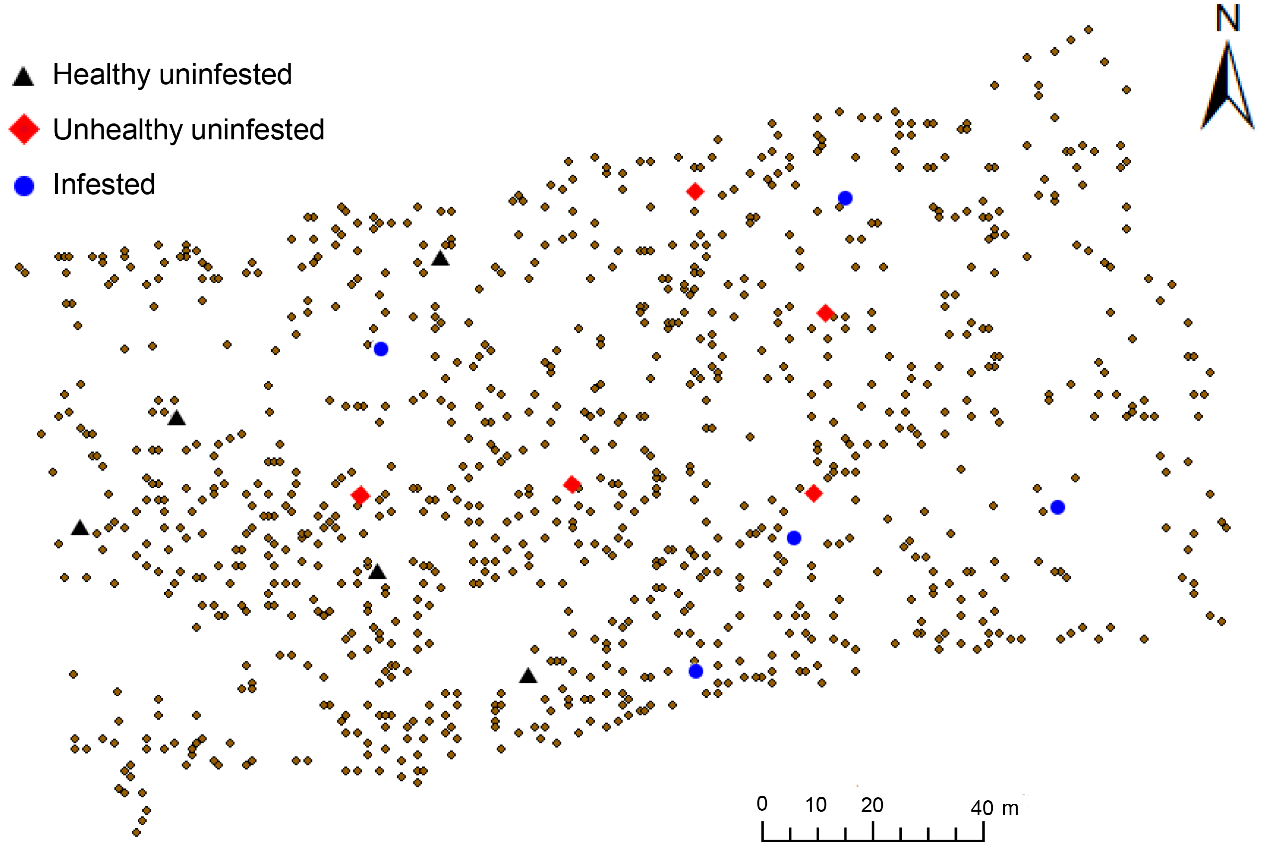
 **Figure S2**. The distance locations of the fifteen trees. Black triangles, healthy uninfested; Red squares, unhealthy uninfested; Blue circles, the woodwasp *Sirex noctilio* infested Mongolian pine trees.

Table S1. The occurrence of *Diplodia sapinea* on *Pinus* species in the world

| Host | Country | Reference(s) |
| --- | --- | --- |
| *P. radiata* | Spain | Manzanos et al. (2019) |
| *P. tabulaeformis*;  *P. sylvestris* var. *mongolica*;  *P. thunbergii*; *P. massoniana* | China | Halifu et al. (2019);  Tang (2017); Ju (2005) |
| *P. sylvestris* | Germany | Vornam et al. (2019) |
| *P. nigra*; *P. strobus*; *P. radiata*;  *P. ponderosa*; *P. pinaster*;  *P. halepensis* | Bulgaria | [Margarita](https://schlr.cnki.net/home/search?ad=1&sw-input-ath=Margarita%20Georgieva) and [Sianna](https://schlr.cnki.net/home/search?ad=1&sw-input-ath=Sianna%20Hlebarska) (2016) |
| *P. sylvestris* | Latvian | Adamson et al. (2015) |
| *P. patula*  *P. radiata* | South African | Swart et al. (1986) |
| *P. ponderosa* | [Argentina](http://redalyc.org/articulo.oa?id=48118695013) | Mattes Fernández et al. (2010) |
| *P. nigra*; *P. resinosa*;  *P. banksiana* | USA | Blodgett et al. (2003);  Blodgett et al. (2007);  [Stanosz](https://schlr.cnki.net/home/search?ad=1&sw-input-ath=G.%20R.%20Stanosz) et al. (1997) |
| *P. nigra* | Italy | Luchi et al. (2004) |
